# The Expression, Purification, and Characterization of a Ras Oncogene (Bras2) in Silkworm (*Bombyx mori*)

**DOI:** 10.1155/2013/269609

**Published:** 2013-05-27

**Authors:** Zhengbing Lv, Tao Wang, Wenhua Zhuang, Dan Wang, Jian Chen, Zuoming Nie, Lili Liu, Wenping Zhang, Lisha Wang, Deming Wang, Xiangfu Wu, Jun Li, Lian Qian, Yaozhou Zhang

**Affiliations:** ^1^College of Life Sciences, Zhejiang Sci-Tech University, Hangzhou 310018, China; ^2^Zhejiang Provincial Key Laboratory of Silkworm Bioreactor and Biomedicine, Hangzhou 310018, China; ^3^School of Pharmacy, Xuzhou Medical College, Xuzhou 221004, China; ^4^Department of Obstetrics and Gynecology, Yong Loo Lin School of Medicine, National University Health System, Singapore 119228; ^5^Agilent Technologies Singapore, Singapore 117681

## Abstract

The Ras oncogene of silkworm pupae (Bras2) may belong to the Ras superfamily. It shares 77% of its amino acid identity with teratocarcinoma oncogene 21 (TC21) related ras viral oncogene homolog-2 (R-Ras2) and possesses an identical core effector region. The mRNA of* Bombyx mori *Bras2 has 1412 bp. The open reading frame contains 603 bp, which encodes 200 amino acid residues. This recombinant BmBras2 protein was subsequently used as an antigen to raise a rabbit polyclonal antibody. Western blotting and real-time PCR analyses showed that BmBras2 was expressed during four developmental stages. The BmBras2 expression level was the highest in the pupae and was low in other life cycle stages. BmBras2 was expressed in all eight tested tissues, and it was highly expressed in the head, intestine, and epidermis. Subcellular localization studies indicated that BmBras2 was predominantly localized in the nuclei of Bm5 cells, although cytoplasmic staining was also observed to a lesser extent. A cell proliferation assay showed that rBmBras2 could stimulate the proliferation of hepatoma cells. The higher BmBras2 expression levels in the pupal stage, tissue expression patterns, and a cell proliferation assay indicated that BmBras2 promotes cell division and proliferation, most likely by influencing cell signal transduction.

## 1. Introduction

Ras family small GTPases play essential roles in a variety of cellular responses including cell proliferation, differentiation, survival, transformation, and tumor development [1–4]. The family has approximately 20 members in mammals [5, 6], and as many as 36 Ras family genes have been identified in humans [7] with evolutionarily conserved orthologs in *Drosophila*, *C. elegans*, *S. cerevisiae*, *S. pombe*, *Dictyostelium, *and plants [8]. This family includes the classical Ras proteins (H-Ras, N-Ras, K4A-Ras, and K4B-Ras), the R-Ras proteins (R-Ras, TC21/R-Ras2, and M-Ras/R-Ras3), the Rap proteins (Rap1A, Rap1B, Rap2A, and Rap2B), and the Ral proteins (RalA and RalB) [9]. The first identified classical Ras (hereafter simply referred to as Ras) proteins have been studied most intensively. It was shown that Ras transduces signals from receptor-type tyrosine kinases to downstream effectors and thereby controls the proliferation and differentiation of various cell types [10]. Ras proteins function as molecular switches and are controlled by a regulated GDP/GTP cycle. Guanine nucleotide exchange factors (GEFs, e.g., SOS and mCDC25/GRF) promote the formation of active, GTP-bound Ras, whereas GTPase-activating proteins (GAPs, p120 and NF1 GAP) promote the formation of inactive, GDP-bound Ras [11].

Like Ras, other members of the Ras superfamily are also believed to function as molecular regulatory switches that control a spectrum of diverse cellular processes [12]. Ras-related proteins share significant similarities in molecular weight (20 to 25 kDa) and sequence identity (30 to 55%) with Ras proteins [13]. Despite possessing strong structural and biochemical similarities with Ras proteins, only a limited number of Ras-related proteins have been shown to exhibit transforming potential [14]. Apart from the classical Ras proteins, the only other member of the Ras subfamily of GTPases found to be mutated in human cancers was TC21 (also called R-Ras2) [15].

The aim of this study is to elucidate ras oncogene (*Bras2*) properties and to characterize the *Bras2 *present in the silkworm *Bombyx mori*. The cDNA of *BmBras2* from *Bombyx mori* consists of 1,412 bp. The open reading frame (ORF) contains 603 bp, encoding 200 amino acid residues with a predicted molecular weight of 22.9 kDa and theoretical isoelectric point (pI) of 6.62. Its accession number in GenBank is AB206960. In this paper, we report the cDNA cloning, expression, purification, and characterization of silkworm Bras2 for the first time. We found that silkworm Bras2 shares 77% of its amino acid identity with TC21 and may be involved in the regulation of normal cell growth. This study lays a good foundation for further research on the function of this protein.

*Bombyx mori *has a well-studied genetic background and high developmental synchronization. Because it is susceptible to nuclear polyhedrosis virus and easy to breed at a large scale, *B. mori* has been used as a bioreactor to produce recombinant proteins with the *B. mori *nucleopolyhedrovirus (BmNPV) expression system [16]. One of the major advantages of the BmNPV expression system is that it can be used to produce relatively large quantities of posttranslationally modified heterologous proteins. This expression system is inexpensive, convenient and has a high production level, so it has been widely used to express recombinant proteins.

## 2. Materials and Methods

### 2.1. Animals, Tissues, and Bm5 Cells

The *B. mori* strain in this study was the progeny of Qiufeng × Baiyu. Silkworms were reared on mulberry leaves under standard conditions. Heads, intestines, epidermises, silk glands, fat bodies, malpighian tubules, ovaries, and testes were dissected from the fifth instar larvae, frozen immediately in liquid nitrogen, and stored at −80°C. The fifth instar larvae, pupae, moths, and nascent eggs were also frozen in liquid nitrogen and stored at −80°C. Bm5 cells were seeded at 1 × 10^5^ cells per flask in culture flask and cultured for three days at 37°C in a 5% CO_2_ incubator. The medium was removed and the Bm5 cells were collected for western bolt with anti-BmBras2.

### 2.2. Construction of a Recombinant Plasmid

The cDNA, which was previously constructed from metaphase pupae by our laboratory [17], was used as a template to amplify the coding region of BmBras2 by polymerase chain reaction (PCR). We designed gene-specific primers on the basis of the *BmBras2 *cDNA sequence, which included restriction enzyme sites for *Eco*RI and *Hind*III. The sequences of the gene-specific primer (GSP) were 5′-GGGAATTCATGTCTCGAGCAGGCGACAGAC-3′ and 5′-GGGAAGCTTTTACAGGATGGTGCACTTC-3′. The PCR cycle conditions were one cycle at 94°C for 5 min, then 30 cycles of denaturing at 94°C for 1 min, followed by an annealing step at 53°C for 50 s, then an extension step at 72°C for 1 min and one additional extension cycle at 72°C for 10 min using a *Taq* DNA polymerase Kit (Promega, USA). The PCR products were purified using a PCR Rapid Purification Kit (BioDev-Tech, China). After digestion with* Eco*RI and *Hind*III, the amplicons were subcloned into expression vector pET-28a using T4 DNA ligase (Promega, USA) and then transformed into *E. coli* TG1 cells (which are maintained in our laboratory) for screening purposes. Positive colonies in which the *BmBras2* gene was successfully integrated into the plasmid were identified by double plasmid digestion and subsequently sequenced by an ABI PRISM 3130-XL/A automated sequencer (applied biosystems).

### 2.3. Sequence Analysis

A nucleotide and protein sequence similarity analysis was carried out at GenBank using BLASTN (in the EST, other database) and BLASTP (in all nonredundant databases) algorithms. The deduced amino acid sequence was analyzed with the Expert Protein Analysis System (http://www.expasy.org/). Multiple sequence alignments of the Bras2 and R-Ras family were conducted using the Clustal W program in Bioedit software. The protein conformation was modeled by SWISS-MODEL (http://swissmodel.expasy.org/) and viewed in the Swiss PDB Viewer [18].

### 2.4. Expression and Purification of *BmBras2*

The recombinant expression plasmid pET-28a (+)-*BmBras2* was transformed into* E. coli* Rosetta (DE3) (which is maintained in our laboratory). Bacterial expression cultures were incubated at 37°C in LB medium containing kanamycin (50 *μ*g/mL) and chloramphenicol (50 *μ*g/mL) until an A_600_ of 0.5 was reached. Recombinant protein expression was induced by the addition of IPTG (Sanland-chem, USA) at a final concentration of 0.1 mM. Following 4 h of incubation at 37°C, bacterial culture was harvested by centrifugation and frozen at −20°C. Frozen bacterial pellets were thawed and resuspended in lysis buffer and then lysed by pulsed sonication with a cell disruptor (SCIENTZ-IID) on ice. The lysates were centrifuged at 14,000 × g for 20 min at 4°C. The supernatant was collected and filtered through a 0.45 *μ*m filter (Millipore, USA). Nickel metal affinity resin columns were used for rBmBras2 single-step purification [19], and the protein purity was examined by SDS-PAGE as described by Laemmli [20]. The purity was also examined by HPLC, which showed a fusion protein purity of over 98%.

### 2.5. Molecular Weight Determination

The molecular weight of rBmBras2 was assessed by SDS-PAGE using 12% SDS gels. The molecular weight was also determined by 4700 MALDI-TOF/TOF mass spectrometry [21] (ABI, USA) using default settings for laser energy and TOF parameters.

### 2.6. Protein Extraction

The fifth instar larvae, pupae, moths, eggs, and tissues isolated from fifth instar larvae were ground to a powder in liquid nitrogen. The powders were suspended in protein dissolution buffer and then incubated for 30 min on ice. Homogenates were centrifuged at 12,000 × g for 15 min at 4°C. The protein concentrations of all samples were quantified before SDS-PAGE analysis using a Bradford assay, and total protein loading was normalized to an equal amount with alpha tubulin.

### 2.7. Western Blot Analyses

Whole protein extracts from each tissue and Bm5 cells were separated by 12% SDS-PAGE and then electrotransferred to PVDF membranes (Millipore). Membranes were incubated in blocking buffer containing 5% skim milk and 0.05% Tween 20 in TBS (TBST; pH 7.5) at room temperature for 1 h or 4°C overnight and were then incubated with polyclonal antibody (diluted to 1/1000) in blocking buffer at room temperature for 1 h or 4°C overnight, then washed with 0.05% TBST three times for 5 min per wash and detected with HRP-labeled anti-rabbit IgG (Dingguo Biotechnology) and DAB. The preparation of polyclonal antibody for anti-rBmBras2 was described by Sheng et al. [22].

### 2.8. RNA Extraction

Total RNA was extracted from the various silkworm developmental stages and the tissues of fifth instar larvae using Trizol reagent (Invitrogen) according to the manufacturer's instructions. Contaminating genomic DNA (gDNA) was removed by adding DNase I (Invitrogen). RNA purity was determined with a UV spectrophotometer. The UV_260_/UV_280_ ratios were between 1.8 and 2.1 for all analyzed RNA samples. The concentration of total RNA was determined by measuring the absorbance at 260 nm with an ND-1000 spectrophotometer (Bio-Rad, USA).

### 2.9. Primer Design and Real-Time PCR

Real-time PCR primers were designed using Primer Premier 5.0 software. The primer pairs for BmBras2 were as follows: upstream primer, 5′-TCCTGCTGGTCTTCTCCGTG-3′ downstream primer, 5′-GACACCACTCGCTGCGTTTC-3′; 18S rRNA, upstream primer, 5′-CGATCCGCCGACGTTACTACA-3′; and downstream primer, 5′-GTCCGGGCCTGGTGAGATTT-3′. Real-time PCR was performed with an ABI Prism 7300 Sequence Detection System (Applied Biosystems) under the following PCR conditions: one cycle at 95°C for 30 s, followed by 40 cycles at 95°C for 5 s and 60°C for 31 s. A SYBR PrimeScript RT-PCR Kit II was used for the real-time PCR. Each reaction was performed in triplicate in a 96-well plate along with the endogenous 18S rRNA control gene. At the end of the real-time PCR cycles, a dissociation curve was performed to check for the presence of nonspecific dsDNA SYBR Green hybrids, such as primer dimers. Data analysis was performed with ABI Prism 7300 SDS Software V1.3.1 (Applied Biosystems, USA). The target gene expression levels were normalized against the 18S rRNA gene expression level. The relative expression level was calculated using 2^−ΔΔCT^. (Where ΔCT = CT (*BmBras2*) − CT (18S rRNA) for different stages or tissues; ΔΔCT = ΔCT for different stages or tissues − ΔCT maximum).

### 2.10. Subcellular Localization

Bm5 cells were seeded in a special confocal microscope dish (Bio-line Instruments). After 12 h, the culture medium was removed. Cells were rinsed twice with 1 mL phosphate-buffered saline (PBS), and then they were fixed in 3.7% formaldehyde at 25°C for 10 min. The cells were blocked with 3% BSA at 37°C for 2 h. After that, the cells were incubated with anti-BmBras2 IgG antibody (dilution 1/1000) at 4°C for 12 h (cells incubated with negative serum without the antibody served as the control). After three washes in PBST (PBS+0.05% Tween-20, 10 min each), cells were incubated with goat anti-rabbit antibody (dilution, 1/2000, Cy3 labeled, Promega) and DAPI (4′-6-diamidino-2-phenylindole, at a dilution of 1/2,000) at 37°C for 2 h. Following three washes with PBST (10 min each), the cells were analyzed with a Nikon ECLIPSE TE2000-E confocal microscope with EZ-C1 image analysis software.

### 2.11. Cell Proliferation Assay

One hundred microliters of cell suspension (approximately 2 × 10^3^ hepatoma cells) was added to each well of a 96-well plate and cultured for one day at 37°C in a 5% CO_2_ incubator. Ten microliters of rBmBras2 protein was pipetted into each well, and then the hepatoma cells were divided into a control group (0 mg/mL) and test groups of rBmBras2 at different concentrations (1.5, 5, 20, 40 *μ*g/mL) with 6 replicates per group. The hepatoma cells were then cultured for 3 more days in the cell incubator. Ten microliters of Cell Counting Kit-8 (CCK-8) solution was transferred to each well in the 96-well plate and incubated for approximately 1 hour. All samples were measured with a Universal Microplate Reader ELX-800 (Bio-Tek, USA) at a wavelength of 450 nm for measurements and 630 nm for references. All results were presented as $\overset{-}{x}\pm s$, and a Student's *t*-test was used for statistical analysis, with statistical significance defined as *P* < 0.05 or *P* < 0.01.

## 3. Results

### 3.1. Biological Information about *BmBras2*

**Figure 1:**
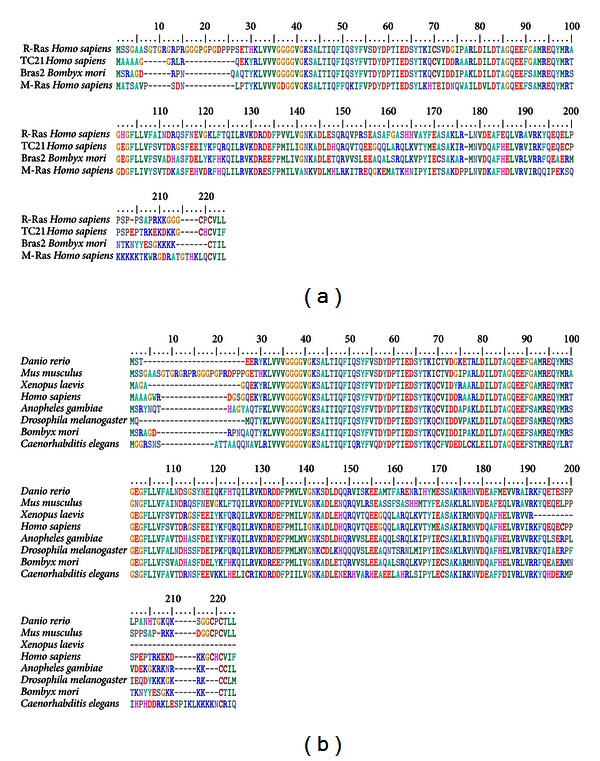
(a) Alignment of amino acid sequence of BmBras2 with members of the R-Ras superfamily from *Homo sapiens*. BmBras2 shares 69% of its amino acid identity with R-Ras, 77% with TC21 (R-Ras2) and 59% with M-Ras (R-Ras3). (b) Alignment of BmBras2 amino acid sequence with homologous proteins from different species.

The complete mRNA of the *BmBras2* gene was 1412 bp in length. This length includes a 5′-terminal untranslated region (UTR) of 122 bp, a 3′-terminal UTR of 687 bp with a canonical polyadenylation signal sequence of AATAAA and a poly(A) tail, and an open reading frame (ORF) of 603 bp encoding a polypeptide of 200 amino acids. The BmBras2 protein had a predicted molecular weight of 22.9 kDa and a theoretical isoelectric point of 6.62. The conserved domain of BmBras2 shared specific similarities with the M-Ras/R-Ras-like subfamily, and this subfamily contains R-Ras2/TC21, M-Ras/R-Ras3, and related members of the Ras family. The R-Ras family of Ras-related proteins contains R-Ras, TC21 (R-Ras2), and M-Ras (R-Ras3). We employed a Clustal W program in Bioedit software to generate multiple alignments of the R-Ras family members and BmBras2, and significant similarities were detected between members of the R-Ras family and BmBras2. BmBras2 shares 77% of its amino acid identity with TC21 (R-Ras2) and also includes the identical core effector regions (Figure 1(a)). BmBras2's homology with ras-related proteins from other species is very high, with 76% shared amino acid identity with a Ras-related protein R-Ras2 precursor of Mus musculus and 78% amino acid identity with the Ras oncogene at 64B of *Drosophila melanogaster* (Figure 1(b)). These analyses indicated that the BmBras2 of silkworm pupae may belong to the Ras superfamily.

### 3.2. rBmBras2 Expression and Purification in *E. coli*

**Figure 2:**
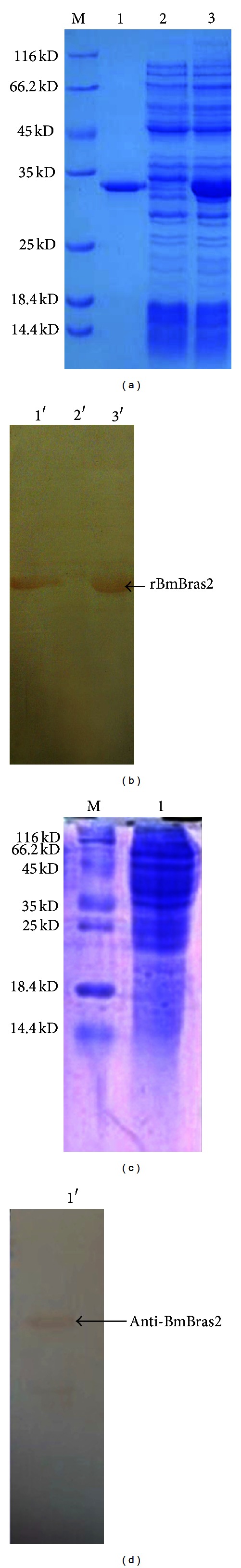
(a) The expression and purification of recombinant BmBras2 were analyzed by SDS-PAGE. (b) The expression and purification of recombinant BmBras2 were analyzed by Western blotting. M, protein mass marker; 1, purified recombinant protein by Ni-NTA superflow cartridges; 2, the lysate of *E. coli* Rosetta with pET-28a (+)-*BmBras2* without induction; 3, the lysate of *E. coli* Rosetta with pET-28a (+)-*BmBras2* after IPTG induction. (c) The lysate of Bm5 was analyzed by SDS-PAGE. (d) The Bm5 cells were collected for western bolt with anti-BmBras2. (Bm5 cells were seeded at 1 × 10^5^ cells per flask in culture flask and cultured for three days at 37°C in a 5% CO_2_ incubator.) M, protein mass marker; 1, the lysate of Bm5 cells.

A PCR fragment containing the complete open reading frame of mature BmBras2 was inserted into pET-28a by ligation at the *Eco*RI and *Hind*III restriction enzyme sites. The expressed product was a recombinant protein with a 6 × His tag. The fusion protein His-BmBras2 was successfully expressed in *E. coli* and purified using nickel metal affinity resin columns. A molecular weight of 26,690.0 D was determined by MALDI-TOF/TOF analysis (data not shown) and is in agreement with the calculation using amino acid composition (22879.1 D + 3825.2 D = 26,704.3 D). The polyclonal antibody was prepared by subcutaneously immunizing male New Zealand white rabbits. The titer of the polyclonal antibody was more than 1 : 12800 when measured by indirect ELISA (data not shown). In Western blot analyses (Figure 2), the polyclonal antibody recognized recombinant His-BmBras2 protein.

### 3.3. Expression Analysis and Tissue Distribution of *BmBras2*

**Figure 3:**
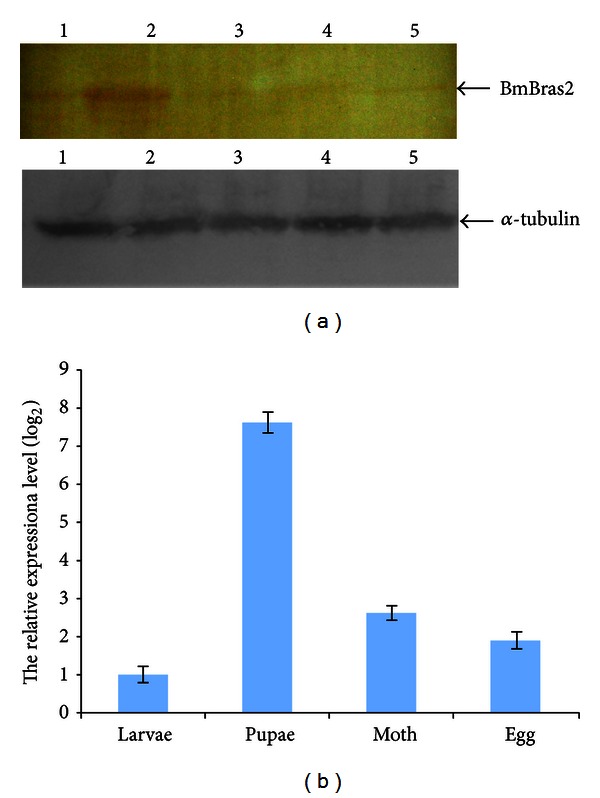
The expression analysis of BmBras2 during different silkworm developmental stages. (a) The expression levels of BmBras2 by western blotting; 1, fifth instar larvae; 2, pupae; 3, moth; 4, egg; 5, purified rBmBras2. (b) The relative expression levels of BmBras2 analyzed by real-time PCR. The relative expression level was calculated by using 2^−ΔΔCT^, where ΔΔCT = (CT, *BmBras2*-CT, 18S rRNA) for different stages and (CT, *BmBras2*-CT, 18S rRNA) pupae.

**Figure 4:**
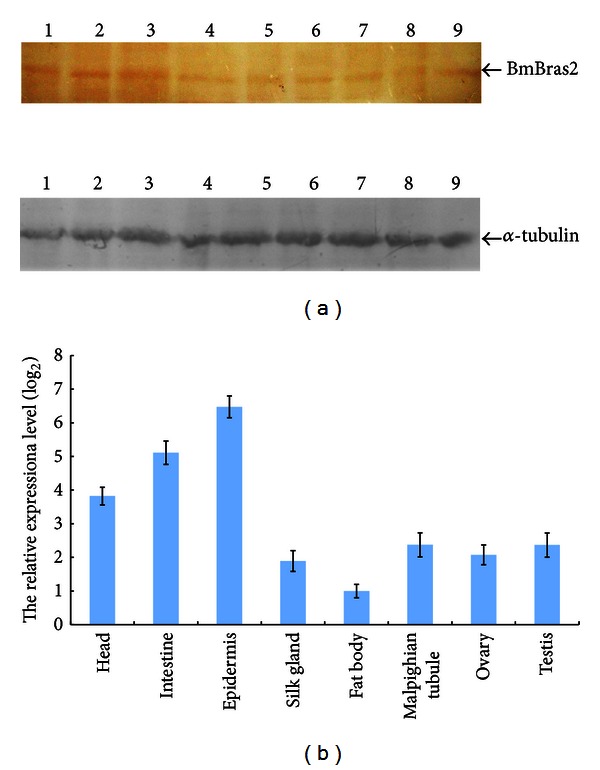
Distribution of BmBras2 in different fifth instar larvae tissues. (a) tissue distributions of BmBras2 by western blotting; 1, head; 2, intestine; 3, epidermis; 4, silk gland; 5, fat body; 6, malpighian tubule; 7, ovaries; 8, testis; 9, purified rBmBras2. (b) tissue distributions of BmBras2 analyzed by real-time PCR. Sg, silk gland; Fb, fat body; Mt, malpighian tubule. The relative expression level was calculated by using 2^−ΔΔCT^ here ΔΔCT = (CT, *BmBras2*-CT, 18S rRNA) for different tissues and (CT, *BmBras2*-CT, 18S rRNA) fat body.

There is no information regarding the expression patterns of BmBras2 from EST sources. We therefore employed SYBR Green real-time PCR and western blot analyses to quantify *BmBras2* gene expression levels during different silkworm developmental stages and tissue distribution in fifth instar larvae [23]. For Western blot analyses, we used the polyclonal rabbit antibody (Anti-rBmBras2) to analyze the protein expression levels throughout four silkworm developmental stages and from eight tissues of the fifth instar larvae. For the real-time PCR analyses, we used a constitutively expressed gene, namely, 18S rRNA, as an internal control. For the *BmBras2* gene and 18S rRNA of silkworm, dissociation curves indicated proper amplification of the intended targets at the corresponding melting temperatures. *BmBras2* expression levels during different silkworm developmental stages are shown in Figure 3. BmBras2 was expressed throughout four developmental stages, and its expression was higher in the pupal stage and lower in others such as the fifth instar larvae, moth, and nascent egg. The real-time PCR results were consistent with the western blot analyses. Tissue distributions of BmBras2 in the fifth instar larvae are shown in Figure 4(a), with alpha-tubulin as an internal control. According to real-time PCR analysis, BmBras2 was expressed in all eight tissues (Figure 4(b)), and it was highly expressed in the head, intestine, and epidermis. Real-time PCR results were basically consistent with the western blot analyses (Figure 4(a)). Western blot analysis of Bm5 lysate illustrated that there was a clear target band near 35 kDa band of the maker, while others are not obvious, which show good specificity of the prepared antibodies. 

### 3.4. Subcellular Localization of *BmBras2*

**Figure 5:**
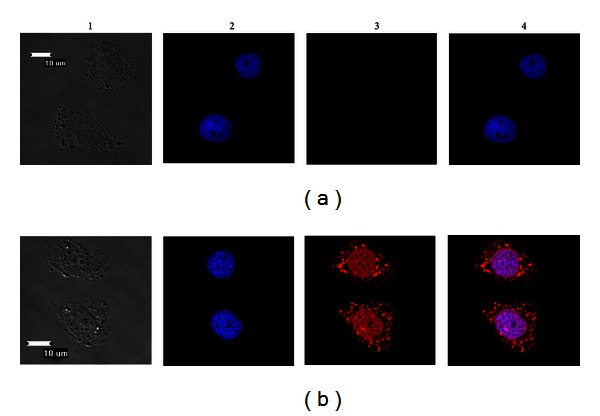
Subcellular localization of BmBras2. (a) Negative result amplified by 40 × 2.5; (b) positive result amplified by 40 × 2.5; 1, visible light images; 2, DAPI fluorescence images; 3, Cy3 fluorescence images; 4, mixed images.

Bm5 cells were used to investigate the subcellular localization of endogenous BmBras2. Immunostaining with polyclonal rabbit anti-rBmBras2 antibody indicated that BmBras2 localized in both the cytoplasm and nucleus. The fluorescence intensity was predominantly stronger in the nucleus than in the cytoplasm, which indicated that BmBras2 is mainly localized in the nuclei of Bm5 cells (Figure 5).

### 3.5. rBmBras2 Effects on Cell Proliferation

**Figure 6:**
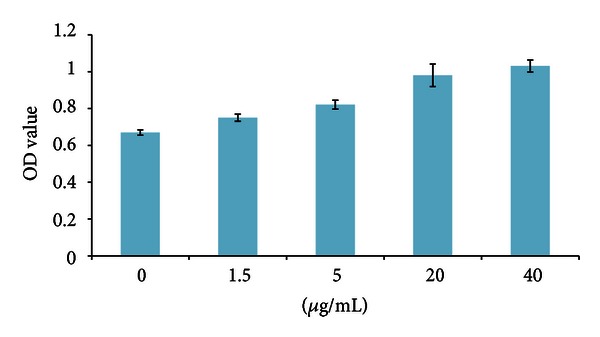
rBmBras2 effects on PLC proliferation promotion.

PLC cells were treated with rBmBras2 for approximately 3 days and then measured at a wavelength of 450 nm with CCK-8. According to Figure 6, rBmBras2 proliferated on PLC in a dose-dependent manner. In other words, the effect of cell proliferation increased with an increasing concentration of rBmBras2 (*P* < 0.05). Meanwhile, the same assay was performed using HepG2 cells. The result showed that rBmBras2 can also promote the proliferation of HepG2 cells with the same effect as the PLC cells (data not shown). 

## 4. Discussion

Ras superfamily GTPases function as GDP/GTP-regulated molecular switches [24]. They share a set of conserved G box GDP/GTP-binding motif elements beginning at the N-terminus as follows: G1, GXXXXGKS/T; G2, T; G3, DXXGQ/H/T; G4, T/NKXD; and G5, C/SAK/L/T [25]. We aligned the amino acid sequence of BmBras2 in the NCBI database and found that its conserved domain also contained G box GDP/GTP-binding motif elements. This protein showed specific similarities with an M-Ras/R-Ras-like subfamily, and it belongs to the Ras-like GTPase superfamily. Many members of the Ras superfamily of GTPases have been implicated in hematopoietic cell regulation, with roles in growth, survival, differentiation, cytokine production, chemotaxis, vesicle trafficking, and phagocytosis [6]. However, it is becoming increasingly evident that different members of the Ras subfamily may have different biological functions that depend not only on differences in their affinities to regulators or effectors but also on their precise subcellular localization [6].

The silkworm undergoes a complete metamorphosis, and its life cycle has four developmental stages, that is, egg, larvae, pupae, and moth. During these four stages, there are substantial changes in external morphology, physiological function, and biological characteristics. Although the insects seemed to be superficially very quiet during periods such as the egg stage, pupal stage, silking stage, and the molting stage, there are drastic internal changes going on; at the surface in the pupal stage, however, there is intensive internal organizational dissociation and histogenesis. These changes prepare the silkworm for the mating and oviposition of the adult stage. We analyzed the expression of BmBras2 during different developmental stages, and we found that the expression level of our target protein was the highest during the pupal stage and lower in the fifth instar larvae, moths, and nascent eggs. Because intense tissue differentiation and dissociation happen during the pupal stage, we predicted that BmBras2 might play an important role in this process.

Silkworm growth is a comprehensive embodiment of system and organ growth, and the growth of systems and organs is the embodiment of cell growth. There are three ways for cells to grow, as follows: (1) cell division, in which the growth pattern mainly relies on increasing the number of similar cells, but the basic cell size remains unchanged, as in sperm cells; (2) cell volume increase, with cell division mainly appearing during the embryonic development stage, but in the larval stage, the growth of entire organs and tissues is implemented only by increasing the cell size, as in silk glands; (3) cell division and cell volume increase, which include most of tissues and organs, such as epithelial cells. To learn more about the distribution and related functions of BmBras2 protein in various tissues, we measured mRNA and protein expression levels. The results of both experiments are largely consistent with one another, and BmBras2 is highly expressed in the head, intestine, and epidermis. High expression levels of tissue BmBras2 are essential for silkworms to complete their growth and development (that is, for cell division and proliferation). We have therefore proposed that BmBras2 protein is involved in cell cycle regulation, and it can promote cell division and proliferation through cell signal transduction, which is needed for tissue differentiation and dissociation.

Subcellular localization is closely related to protein function. Immunofluorescence analysis indicated that BmBras2 is mainly localized in the nuclei of Bm5 cells and partly in the cytoplasm. It was thus hypothesized that BmBras2 may be involved in cell cycle regulation, and this result will lay a foundation for further study of BmBras2 protein function.

TC21 is known to be a powerful oncogene [7], and constitutive TC21 activity induces cell proliferation and transformation [9]. TC21 overexpression in hepatocellular carcinoma (HCC) has been positively correlated to tumor size. Sequence analysis results suggest that Bmbras2 shares a high homology with TC21. To investigate whether BmBras2 can stimulate hepatoma cell proliferation, we conducted a cell proliferation assay. The results showed that rBmBras2 protein can promote PLC and HepG2 cell proliferation, but the cell proliferation mechanism caused by rBmBras2 protein is unclear and requires further study.

The silkworm is a widely used model organism. Basic research on the silkworm Bras2 protein location and biological functions will provide an important basis for further study of this protein's physiological role as well as that of other silkworm protein families.
